# Cell Death and Heart Failure in Obesity: Role of Uncoupling Proteins

**DOI:** 10.1155/2016/9340654

**Published:** 2016-08-23

**Authors:** Angélica Ruiz-Ramírez, Ocarol López-Acosta, Miguel Angel Barrios-Maya, Mohammed El-Hafidi

**Affiliations:** Departamento de Biomedicina Cardiovascular, Instituto Nacional de Cardiología Ignacio Chávez, Juan Badiano No. 1, Colonia Sección XVI, Tlalpan, 14080 Ciudad de México, DF, Mexico

## Abstract

Metabolic diseases such as obesity, metabolic syndrome, and type II diabetes are often characterized by increased reactive oxygen species (ROS) generation in mitochondrial respiratory complexes, associated with fat accumulation in cardiomyocytes, skeletal muscle, and hepatocytes. Several rodents studies showed that lipid accumulation in cardiac myocytes produces lipotoxicity that causes apoptosis and leads to heart failure, a dynamic pathological process. Meanwhile, several tissues including cardiac tissue develop an adaptive mechanism against oxidative stress and lipotoxicity by overexpressing uncoupling proteins (UCPs), specific mitochondrial membrane proteins. In heart from rodent and human with obesity, UCP2 and UCP3 may protect cardiomyocytes from death and from a state progressing to heart failure by downregulating programmed cell death. UCP activation may affect cytochrome c and proapoptotic protein release from mitochondria by reducing ROS generation and apoptotic cell death. Therefore the aim of this review is to discuss recent findings regarding the role that UCPs play in cardiomyocyte survival by protecting against ROS generation and maintaining bioenergetic metabolism homeostasis to promote heart protection.

## 1. Introduction

Obesity is characterized by oxidative stress, mitochondrial damage, and cardiac dysfunction by ectopic triglycerides (TGs) accumulation in myocardium as a result of elevated circulating free fatty acids (FFAs) delivered from adipose tissue and leading to myocardial steatosis [1]. Lipid accumulation in myocardium may enhance mitochondrial ROS generation and oxidative stress which increase the risk of heart failure in obese and diabetic subjects [2]. The early stage of heart failure in obesity is characterized by an overexpression of UCP2 or UCP3 in response to increased FFA and mitochondrial ROS generation and induced mild uncoupling of oxidative phosphorylation without affecting ATP level [3]. However, upon progression of obesity to morbid obesity, FFA concentration increases more than the level found during early stage of obesity and induces a strong uncoupling of mitochondrial oxidative activity resulting in a significant drop in cardiac ATP levels and cell death and finally heart failure [4, 5]. Mitochondria are essential for cell survival by producing energy and maintaining redox status. Moreover, mitochondrion is an organelle where most of ROS generation in the whole cell occurs [6, 7]. Under physiological conditions it has been estimated that 1 to 2% of the oxygen consumed in mitochondria becomes superoxide anion (O_2_^•−^) that substantially contributes to total cellular ROS production [8]. In pathological conditions such as metabolic syndrome and obesity, excessive fatty acid *β*-oxidation, lipotoxicity, and deficient antioxidative capacity are involved in excessive production of reactive oxygen that induce damage to myocardial structure and function [9, 10]. Generally against uncontrolled ROS generation and oxidative damage, cell develops different defense systems depending on the source of ROS within cell. At the mitochondrial level there is an overexpression of uncoupling proteins (UCPs) whose function is not yet well established. Nevertheless available data point to a general role of UCP1, UCP2, and UCP3 in the regulation of cellular fuel metabolism and ROS production through mild uncoupling without affecting oxidative phosphorylation nor ATP biosynthesis [11–15]. This role of UCPs against oxidative stress is supported by genetic UCP deficiency or pharmacologically inhibited models which show an increased oxidative stress and superoxide anion production [16, 17]. In ischemic heart, the genetic deletion of UCP3 leads to heart failure by exacerbating apoptotic cell death [18, 19]. Thus the pharmacological or genetic induction of UCPs may be considered as a strategy to improve and restore the redox homeostasis and to prevent oxidative stress, which causes damage at macromolecular level and leads to cell death by both necrosis and apoptosis in cardiovascular system. UCP overexpression may downregulate programmed cell death and promote cell survival by decreasing mitochondrial ROS generation which contributes to the release of cytochrome c (cyt c) and proapoptotic proteins from mitochondrial intermembrane space into the cytosol [20]. Cyt c release from mitochondria is considered as a key step in the initiation of apoptosis through both intrinsic and extrinsic pathways [21, 22]. Apoptosis, a physiological process that controls cell proliferation, may reduce biomass inducing a deficient function of some organs such as heart and liver. In this review we will give a general description of biochemical and molecular characteristics of UCPs and then we will discuss the role of UCP2 and UCP3 in cell death and heart failure in metabolic disease such as obesity.

## 2. General Characteristics of UCPs

UCPs are located in the mitochondrial inner membrane (MIM) of different organisms such as yeasts, protozoa, plants, birds, and mammals. These proteins show a tripartite structure typical of mitochondrial carrier family, with three homologous sequences of 100 amino acids which form 6 trans-membrane junctions and 3 *α*-helices parallel to the MIM plane on the matrix side [23–25]. There are five isoforms: UCP1, UCP2, UCP3, UCP4, and UCP5. Monomer molecular mass is about 30–34 kD for UCP1, UCP2, and UCP3, while for UCP4 and UCP5 a higher molecular mass was reported ranging from 36 to 38 kDa, and they function in homodimer form [26]. UCP1 is expressed in brown adipose tissue and in thymus [27]. UCP2 is the only ubiquitous isoform, whereas UCP3 is expressed in skeletal and cardiac muscles and shows 54 to 59% homology of amino acid sequence with UCP1; UCP4 has been found in kidney and central nervous system and UCP5, also called brain mitochondrial carrier protein (BMCP1), has been found in brain cells. UCP4 and UCP5 share only 30% identity with the rest of the UCP family [28].

### 2.1. Cardiac UCP2 and UCP3 Regulation

UCP2 and UCP3 are negatively regulated by purine nucleotides such as GTP, ATP, and GDP which are considered as physiological inhibitors [29]. Nevertheless FFAs were suggested as a direct activators of UCPs that increase conductance and dissipate H^+^ electrochemical gradient into heat. UCP2 and UCP3 are also positively regulated by FFA and have a greater affinity for unsaturated fatty acids such as oleic, linoleic, and arachidonic acids than for saturated fatty acids [30]. UCP2 and UCP3 have been reported to be also activated by superoxide anion (O_2_^•−^) as a feedback mechanism [31, 32]. In animal and human with failing heart, the overexpression of both UCP2 and UCP3 is associated with increased plasma FFAs induced by several factors such as diabetes, fasting, and increased fatty acid mobilization from adipose tissue and with thyroid hormone treatment, which all are associated with oxidative or metabolic stress within cardiac cells [33–35]. Diets high in fat and sucrose, thyroid hormone, and specific peroxisome proliferator-activated receptor agonists have been also described to upregulate UCP2 and UCP3 in the heart and promote a mild uncoupling reducing mitochondrial ROS generation and cardiomyocytes apoptosis and thereby ameliorate myocardial function [36–39]. Furthermore UCP2 has been recently described to provide a regulatory mechanism in cell bioenergetics by catalyzing the exchange of malate, oxaloacetate, and aspartate for phosphate and by exporting C4 metabolites from mitochondria to cytosol* in vivo* [40]. Recently UCP2 is proposed to regulate cell proliferation and survival by switching cell metabolism from glucose to fatty acid oxidation to provide cell with necessary ATP [41] playing a relevant role in the cardiomyocyte tolerance to anoxia/reperfusion injury and oxidative stress [42, 43]. Indeed UCP3-knockout hearts generated more reactive oxygen species (ROS) than WT hearts during ischemia/reperfusion and the pretreatment of UCP3-knockout hearts with the pharmacological uncoupling agent such as carbonyl cyanide* p*-(trifluoromethoxy)-phenylhydrazone (FCCP) improved postischemic functional recovery [18].

In animal model of heart failure induced by pressure overload induced by constriction of the ascending aorta, the expression of UCP2 and UCP3 is downregulated and an increase of ROS generation is enhanced to induce cardiac cell hypertrophy [38, 44]. However this model does not involve FFAs-induced upregulation of UCPs that may protect cell from hypertrophy and dysfunction.

### 2.2. Genetic Regulation of UCPs

At the genetic level, an enhancer element of 200 bp has been identified, located approximately 2.4 kb downstream of* ucp-1* gene, and it promotes gene transcription in response to *β*-adrenergic receptors through cAMP pathway [45]. This 200 bp element has binding sites for retinoids, thyroid hormones, peroxisome proliferator-activated receptor-gamma (PPAR*γ*), PPAR*α*, and PGC-1*α* and determines the expression of UCP1, which selectively regulates the thermogenic response in brown adipose tissue [46–48]. Regarding* ucp-2 *and* ucp-3* genes, response elements of PPAR*α*, PPAR*γ*, and PPAR*δ* have been identified in their promoter region [49]. The induction of* ucp* expression by PPARs varies according to the tissue or to its developmental stage. In cardiac and skeletal muscle of neonatal mice, PPAR*α* stimulated by FFAs is the main inducer, whereas in cell lines such as C2C12 and L6 (myoblast cell lines), the main inducer is PPAR*δ*. FFAs also act on PPAR*δ* and PPAR*γ* which form heterodimers with retinoid X receptor (RXR) [50]. In myogenic cells, 9-cis retinoic acid, via PPARs, promotes the expression of* ucp-3* gene which has a proximal region to MyoD (myogenic differentiation protein), to PPAR*δ* and PPAR*α* which form heterodimers with RXR (PPAR/RXR) in response to FFAs accumulation and to thiazolidinediones, PPAR agonists [51]. The PPAR/RXR heterodimer binds to the gene response element and to MyoD as coactivator, which acts not only to promote gene transcription but also to increase sensitivity of PPARs to their ligand [51] such as long chain fatty acids which activate the transcription factor PPAR*α* and induce UCP2 expression in cardiomyocyte [52]. In addition to FFA, growth factors such as insulin growth factors 1 and 2 (IGF1 and IGF2) and fibroblast growth factor 21 (FGF21), a secreted protein from liver, involved in the control of glucose homeostasis, insulin sensitivity, and ketogenesis were recently found to induce the expression of genes encoding mitochondrial UCP2, UCP3, and superoxide dismutase-2 (SOD2) involved in antioxidative pathways in cardiomyocytes in culture preventing inflammation and hypertrophic process [53]. Moreover* ucp-3* genetic deletion promotes mitochondrial dysfunction and increases ROS production and apoptotic cardiomyocytes death during* in vitro* hypoxia and ischemic heart, suggesting that ucp3 might represent an important determinant of infarct size, postischemic cardiac remodeling, and survival [54].

## 3. UCPs, ROS Generation, and Cell Survival

**Figure 1:**
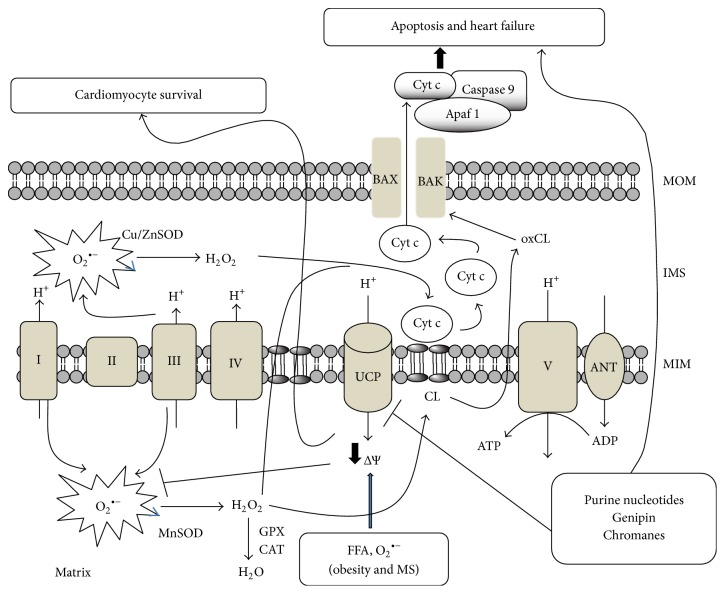
The figure illustrates the role of UCP regulation on cell death or survival. H_2_O_2_, generated from the dismutation of superoxide anion (O_2_^•−^) in both sides of mitochondrial inner membrane (MIM), oxidizes cardiolipin (CL) to produce oxidized CL (oxCL). This reaction is orchestrated with cytochrome c (cyt c) dissociation from MIM. Then free cyt c in IMS (intermembrane space) can cross the MOM (mitochondria outer membrane) by a pore formed by BAX and BAK and induced by oxidized cardiolipin (oxCL). When UCPs are activated, a proton leak (through UCPs) induces a mild uncoupling reducing the formation of O_2_^•−^ and H_2_O_2_ and in turn cyt c release and apoptosis. In addition, cells with deleted UCP or treated with UCP inhibitors such as chromanes or genipin favor ROS generation, cyt c release, cell death, and heart failure.

Several studies evidenced the role of UCPs against oxidative stress and mitochondrial ROS generation induced by different chemical agents such as FFAs, glucose, and lipopolysaccharides or by pathological conditions such as obesity, metabolic syndrome, and type II diabetes [55–60]. In Figure 1, we illustrate the role of UCP in the protection against mitochondrial anion superoxide and H_2_O_2_ generation to induce cardiac cell by both apoptosis and necrosis mechanisms.

Superoxide anion has been described to be generated at seven sites of mitochondrial respiratory complexes and Kreps cycle [61, 62]. However, complexes I and III remain the most important sites of superoxide production. In addition complex III produces superoxide at both sides of mitochondrial inner membrane (MIM). Superoxide anion has a very short half-life and is transformed to H_2_O_2_ by both MnSOD and Cu/ZnSOD found in the matrix and in the intermembrane space (IMS), respectively. The produced mitochondrial H_2_O_2_ is in close proximity to pools of reduced copper and iron (Cu^+^ and Fe^2+^) present in the mitochondrial inner membrane. These transition metal ions catalyze the conversion of H_2_O_2_ through Fenton chemistry to the highly reactive hydroxyl radical (^•^OH) which causes damage to mitDNA, protein complexes, and lipids leading to genetic mutation related to senescence, aging, and cell death [62–64].

### 3.1. ROS Generation and Cell Death

The role of mitochondria in cell survival through a mechanism that involves mitochondrial ROS generation and redox signaling has been largely reviewed by Antico Arciuch et al. [65]. Cell survival or death depends on the amount of ROS generation and it is believed that low levels of ROS may result in activation of signaling pathways and cell survival [66] while excessive mitochondrial ROS production, when not counterbalanced by cell antioxidant defenses, induces cell injury and death [67]. Indeed mitochondrial ROS are pathogenic in metabolic heart disease and cause oxidative modifications of complex I and II proteins by reversible oxidative posttranslational modifications of complex II (subunit B Cysteine100 and Cysteine103) which affect enzymatic catalysis, protein-protein interactions, oxidative phosphorylation, and finally mitochondrial function [68, 69].

To avoid the oxidative damage in mitochondria, H_2_O_2_ is converted to H_2_O by glutathione peroxidase present in the matrix, in a reaction where reduced glutathione is used as electron donor. Moreover, in IMS, H_2_O_2_ generation has been proposed to be located near the sites where cyt c interacts with cardiolipin, a specific phospholipid of MIM, formed by four different fatty acids, mainly linoleic acid, and more susceptible to peroxidation in the presence of H_2_O_2_. Indeed cyt c, in the presence of H_2_O_2_, acquires peroxidase activity and oxidizes cardiolipin and this reaction results in the dissociation of cyt c from mitochondrial inner membrane. The oxidation of cardiolipin reduces cyt c binding and increases the level of soluble cyt c in the IMS. A subsequent release of cyt c occurs by pore formation mediated by proapoptotic Bcl-2 family proteins in mitochondrial outer membranes (MOM), or by Ca^2+^ and ROS-triggered mitochondrial permeability transition, although the latter pathway might be more closely associated with necrosis [61]. In a model of intra-abdominal fat accumulation it was recently suggested that enrichment of cardiolipin with saturated fatty acid, such as palmitic acid, results in a more peroxidation resistant cardiolipin and therefore a reduced dissociation of cyt c from cardiolipin [70]. Once cardiolipin is oxidized and dissociated from cyt c, it becomes enriched in MOM, inducing the insertion of tBid which promotes Bax oligomerization and the formation of pores through which cyt c is released [21, 22]. Thus cyt c release from mitochondria is proposed to occur in two steps: the first step consists in its dissociation from MIM and the second step when cyt c crosses the MOM to cytosol where it participates in the formation of apoptosome. By the change of cardiolipin fatty acid composition found in obesity and by regulating H_2_O_2_ and superoxide anion generation within mitochondria, UCPs may influence cardiolipin peroxidation and cyt c release into cytosol to initiate programmed cell death. This process has been predicted to be associated with uncoupling mitochondrial respiratory chain from ATP biosynthesis which may involve ATP depletion and cell death. Nevertheless, UCP expression was suggested to induce a mild uncoupling without any effect on ATP biosynthesis. In rat model of pressure overload-induced heart failure shows increased mitochondrial capacity to produce ROS increases and proapoptotic gene bax is upregulated with reduced bcl-2/bax ratio to predispose cardiomyocytes to programmed cell death [71–73].

### 3.2. Mild Uncoupling through UCPs and Cell Survival

Mitochondrial ROS generation has been described to be reduced by both mild and strong uncoupling through UCPs, except that strong uncoupling seems to be associated with ATP depletion and probably to cell death by necrosis. However, to date there is no direct evidence that mitochondrial proton leak, mediated by UCPs, lowers ROS production or oxidative damage* in vivo* without affecting ATP biosynthesis. The physiological relevance of UCP-induced mild uncoupling on mitochondrial ROS generation has been discussed recently and it needs more* in vivo* experiments to support its evidence [74]. Nonetheless, a recent report demonstrates that mild uncoupling using the controlled release of oral formulation of 2,4-dinitrophenol (DNP), a mitochondrial protonophore, indirectly produces mild mitochondrial uncoupling and reduces hypertriglyceridemia, insulin resistance, hepatic steatosis, and diabetes in rat models of nonalcoholic fatty liver disease [75]. This report indirectly proposes that mild uncoupling is a probable mechanism by which UCPs protect cell from lipotoxicity and oxidative damage. This suggestion is supported by several* in vitro* experiments which have demonstrated that mild uncoupling mediated by H^+^ flux catalyzed by UCPs has several other physiological implications such as thermogenesis process and the regulation of fatty acid oxidation without altered ATP biosynthesis [76]. The slight uncoupling of the mitochondrial membrane potential by UCP3 or by low concentration of FCCP was demonstrated to be cardioprotective against ischemia/reperfusion (I/R) injury [77, 78].

## 4. Lipotoxicity and UCP2: From Cell Survival to Cell Death

Epidemiological and animal studies have established obesity as an important risk and prognostic factor for heart failure development [79]. In addition, findings from study of obese individuals confirm the existence of a connection between cardiac ectopic fat deposition and cardiac dysfunction [80]. Several mechanisms were proposed to elucidate the nature of the link between obesity and heart failure including the obesity-induced inflammation. However in obesity the major biochemical parameter altered is the chronic elevation of circulating triglycerides (TGs) and FFAs which result in lipid accumulation in cardiac tissues. Fatty acid metabolism is one of the important dynamic and regulated processes in healthy cardiac function. FA supply to the heart is derived from triglycerides hydrolysis in adipose tissue and by the action of lipoprotein lipase within vascular space of lipoprotein particles such as chylomicrons and very low-density lipoprotein (VLDL) to give FFAs the oxidative substrate [81]. In physiological conditions FFAs derived from each of these processes are utilized for energy production through *β*-oxidation, membrane biosynthesis, generation of lipid signaling molecules, posttranslational protein modification, and transcriptional regulation. In obesity with insulin resistance the excess FFAs supply due to high lipolysis activity in adipose tissue contributes to intracellular lipid accumulation due to the impaired utilization of FFAs in the face of continued FFA import or production causing a deregulation of cardiac FFAs oxidation and storage that culminate in TGs accumulation within cardiac myocytes, an early metabolic marker associated with increased left ventricular mass in both obese human and animals model of high-fat diet [79, 80, 82–84]. Several studies from human and animal models of obesity suggest that excessed accumulation of TGs and their hydrolysis products in heart induces oxidative stress and impairs normal cell signaling, causing cellular dysfunction and cell death leading to the pathogenesis of heart failure [81, 85]. In addition, enhanced cardiac FFAs utilization as consequence of lipotoxicity has been described to be associated with reduced cardiac contractile function and efficiency in rodent with obesity and type II diabetes [86, 87].

The situation termed lipotoxicity leads to apoptosis and cell death, by several mechanisms that involve ceramide biosynthesis and changes in membrane phospholipids composition due to the excess saturated fatty acids such as meristic and palmitic acids, which reduce cardiolipin biosynthesis and induce endoplasmic reticulum stress and ROS generation at several sites [88, 89].

## 5. UCPs and Mitochondrial Fatty Acid Oxidation

The overexpression of UCP2 and UCP3 under lipotoxicity conditions suggests that UCPs are required to protect cells from the detrimental consequences of excessive fatty acid metabolism or storage. In skeletal muscle from UCP3 transgenic mice, increased fatty acid oxidation supports the proposed role of UCP3 as an exporter of fatty acid in its anion form from mitochondria to avoid fatty acid accumulation in the matrix [90]. Fatty acid anion results from the hydrolysis of accumulated fatty acyl CoA by mitochondrial acyl-CoA thioesterase-1 (MTE-1) to liberate free CoA required for continued fatty acid oxidation. It is suggested that the MTE1 and UCP3, a PPAR-*α*-regulated gene in cardiac and skeletal muscle, may therefore act together to preserve high rates of fatty acid oxidation in the face of elevated fatty acid availability [91]. In addition, the increased UCP2 and UCP3 in heart, even if being associated with inefficiency of oxidative phosphorylation due to uncoupling, results in increased fatty acid oxidation as fuel utilization [92]. In several pathophysiological conditions such as insulin resistance, diabetes, and postischemia stressed heart, chronic exposure to elevated circulating FFAs leads to increased UCP3 as adaptations that afford protection against the detrimental effect of an acute FFA load [93, 94]. Indeed diabetic heart is characterized by UCP3 overexpression, increased fatty acid oxidation, increased myocardial oxygen consumption, and reduced cardiac efficiency [95].

On the other hand, FFAs are composed by several type fatty acids which are either saturated and contain no double bonds, as palmitic acid (C16:0), monounsaturated which contain only one double bond, such as oleic acid (18:1 n-9), or polyunsaturated (PUFAs) that possess multiple double bonds, typified by *ω*-6 arachidonic acid (20:4 n-6) and *ω*-3 eicosapentaenoic acid (20:5 n-3) and have recently been reviewed to uncouple oxidative phosphorylation, by facilitating proton leakage across the lipid mitochondrial membrane and by decreasing ATP production [96, 97]. In effect they impair electron transport and activate apoptosis and cell death by releasing cytochrome *c* from the inner mitochondrial membrane [98]. In isolated mitochondria, PUFAs have a dual effect on ROS generation depending on their concentration and substrates of direct or forward electron transport through complex I or II of mitochondrial respiratory chain. FFAs induce ROS generation when mitochondria oxidize pyruvate/malate probably by inhibiting complex I. Nevertheless, when mitochondria oxidize succinate [99], the PUFA-reduced effect on ROS generation from mitochondria oxidizing succinate is due to their protonophores property or probably through UCP activation. PUFAs are susceptible to the peroxidation induced by ROS generation. Furthermore UCP2 and UCP3 are suggested to protect mitochondria from peroxidized PUFA by exporting the peroxidized PUFA from the matrix to outside mitochondria through UCP activity [100, 101]. Peroxidized PUFAs are susceptible to degradation giving lipid aldehydes such as 4-hydroxynonenal which increases UCP2 transcription and activates UCP3 dissipating mitochondrial trans-membrane potential and decreasing ROS generation [102, 103].

In regard to saturated FA species such as palmitic acid, there is evidence that they participate in the synthesis of ceramides, a lipid signaling molecule that is well known to induce apoptosis and cell death [104]. In addition saturated fatty acids were also reported to be poor substrates for cardiolipin biosynthesis and lead to a decrease in mitochondrial cardiolipin content that promotes cytochrome c release a key process of apoptosis [105]. The UCP2 overexpression significantly suppresses several markers of cell death, including TUNEL positivity, phosphatidylserine exposure, propidium iodide uptake, and caspase 3 cleavage in cardiomyocytes induced by palmitic acid, and by preventing ROS generation and mitochondrial Ca^2+^ overload [106].

## 6. Mitochondrial Fatty Acid Metabolism and Myocardial Cell Dysfunction

**Figure 2:**
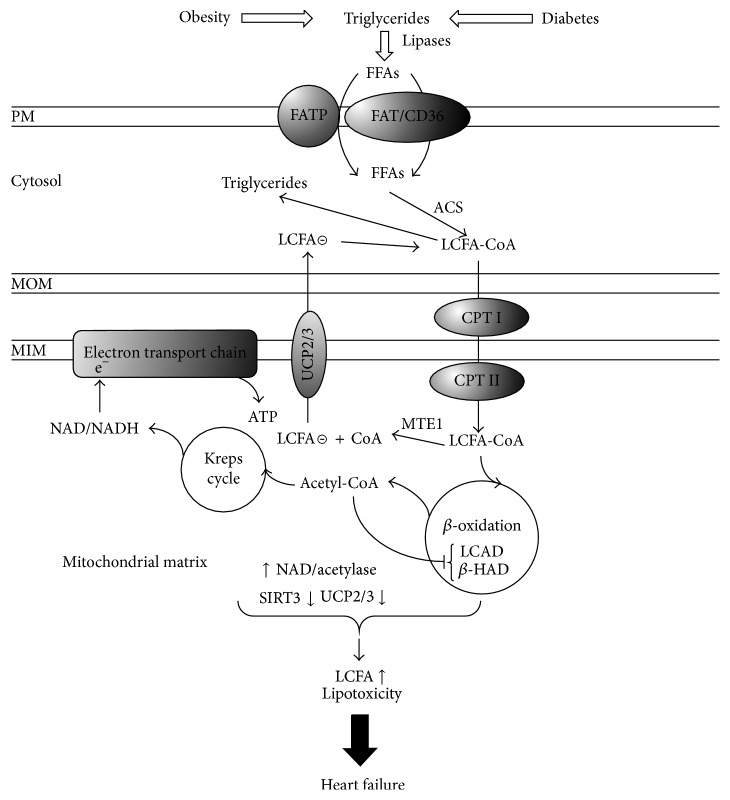
The figure illustrates the role of UCP2/3 to protect cardiomyocytes from LCFA (long chain fatty acid) accumulation in mitochondria and from lipotoxicity induced by increased TGs (triglycerides) and FFAs (free fatty acids) influx to cell. The downregulation of UCPs and SIRT3 and the hyperacetylation of LCAD (long chain acyl-CoA dehydrogenase) and *β*-HAD (*β*-hydroxyacyl-CoA dehydrogenase) lead to the accumulation of LCFA-CoA. Nevertheless MTE1 (mitochondrial acyl-CoA thioesterase-1) liberates free CoA required for continued fatty acid oxidation together with LCFA anion which is exported by UCP2/3 outside mitochondria for its activation by ACS (acyl-CoA synthetase) to comeback mitochondria for the *β*-oxidation. A downregulation of UCP2/3 and MTE1 may result in LCFA accumulation in the matrix and mitochondria dysfunction.

In Figure 2 we illustrated the role of UCP2 and UCP3 in the detoxification of mitochondria from the excess of long chain fatty acid (LCFA) accumulation. Within cells, excess FFAs are physiologically channeled to triglycerides biosynthesis as a protective process against FFAs toxicity [107]; however in pathologic states, lipotoxicity may occur when triglyceride storage is exceeded or when triglyceride pools are hydrolyzed rising unmetabolized FFAs levels which result in alterations of cardiac energetic and mitochondrial fatty acid oxidation defect [108, 109], and this occurs early in obesity and correlates negatively with the fasting plasma free fatty acid concentrations [1].

In a model of obesity (fa/fa Zucker diabetic fatty rats), unmetabolized FFAs, predominantly saturated fatty acids, act directly on mitochondria inducing mitochondrial membrane permeability transition, cytochrome c release, and in turn cardiac cell apoptosis [110]. During fatty acid metabolism, saturated LCFAs bind to carnitine and form long chain acyl carnitine (LCAC) to cross mitochondria inner membrane. In obesity, LCAC such as palmitoyl carnitine accumulates within cardiac cell and causes severe cardiac arrhythmia and dysfunction leading to Ca^2+^ dependent arrhythmia, mitochondrial ROS generation, and decreased adenine nucleotide translocase (ANT) activity [111, 112]. Other intermediary metabolites of fatty acid metabolism such as coenzyme-A (CoA) and its derivatives malonyl-CoA and acetyl-CoA which play an important role in cardiac energy metabolism pathways are involved in altered fatty acid oxidation seen in heart failure. Malonyl-CoA inhibits carnitine palmitoyltransferase-1, a key enzyme involved in mitochondrial fatty acid uptake. Alterations in malonyl-CoA synthesis by acetyl-CoA carboxylase and its degradation by malonyl-CoA decarboxylase are important contributors to the high cardiac fatty acid oxidation rates seen in ischemic heart disease and heart failure in obesity and diabetes [113]. Moreover excess acetyl-CoA contributes to mitochondrial fatty acid *β*-oxidation enzymes acetylation at lysine residue and decreased the activity of long chain acyl-CoA dehydrogenase (LCAD) and *β*-hydroxyacyl-CoA dehydrogenase involved in lipid accumulation and metabolic syndrome development [114, 115]. In addition high-fat feeding downregulates the expression and the activity of SIRT3, deacetylase enzyme leading to LCAD hyperacetylation and decreased activity of fatty acid oxidation in obese heart which result into drop of ATP biosynthesis and heart failure [116]. Thus, a downregulation of UCP2/3 SIRT3 and MTE1 may result in LCFA accumulation in the matrix and may induce mitochondria dysfunction, lipotoxicity, and cell death.

## 7. Summary and Perspectives

Experimental evidence has demonstrated that ROS generation, oxidative stress, and FFA accumulation in myocardium are involved in the molecular processes responsible for cardiomyocyte apoptosis and heart failure and that UCP2 and UCP3 are overexpressed in both obesity and metabolic syndrome to detoxify endogenous produced ROS by mild uncoupling of mitochondria and LCFA accumulation. The mild uncoupling of mitochondria through UCPs or by pharmacological approaches seems to represent a promising target to break the risk factor of obesity-induced heart failure. In addition the combination of safe lifestyle and pharmacologic strategies may help to limit the continued growing of obesity and metabolic syndrome epidemic in the world and improve cardiac dysfunction in obesity by reducing FFAs level.

## Abbreviations

FFAs: Free fatty acids
MS: Metabolic syndrome
ROS: Reactive oxygen species
PPARs: Peroxisomes proliferative activator protein
UCPs: Uncoupling proteins
IMS: Intermembrane space
MIM: Mitochondrial inner membrane
MOM: Mitochondrial outer membrane
RXR: Retinoid X receptor
Cyt c: Cytochrome c
PUFAs: Polyunsaturated fatty acids
LCFA: Long chain fatty acid
LCAD: Long chain
PC: Palmitoyl carnitine.

## References

[1] Kankaanpää M., Lehto H.-R., Pärkkä J. P. (2006). Myocardial triglyceride content and epicardial fat mass in human obesity: relationship to left ventricular function and serum free fatty acid levels. *The Journal of Clinical Endocrinology & Metabolism*.

[2] Joel D. (2015). The mitochondria in diabetic heart failure: from pathogenesis to therapeutic promise schilling. *Antioxidants & Redox Signaling*.

[3] Laskowski K. R., Russell R. R. (2008). Uncoupling proteins in heart failure. *Current Heart Failure Reports*.

[4] Rider O. J., Cox P., Tyler D., Clarke K., Neubauer S. (2013). Myocardial substrate metabolism in obesity. *International Journal of Obesity*.

[5] Murray A. J., Anderson R. E., Watson G. C., Radda G. K., Clarke K. (2004). Uncoupling proteins in human heart. *The Lancet*.

[6] Ježek P., Hlavatá L. (2005). Mitochondria in homeostasis of reactive oxygen species in cell, tissues, and organism. *International Journal of Biochemistry and Cell Biology*.

[7] Kowaltowski A. J., de Souza-Pinto N. C., Castilho R. F., Vercesi A. E. (2009). Mitochondria and reactive oxygen species. *Free Radical Biology and Medicine*.

[8] Cadenas E., Davies K. J. A. (2000). Mitochondrial free radical generation, oxidative stress, and aging. *Free Radical Biology and Medicine*.

[9] Liu S., Geng B., Zou L. (2015). Development of hypertrophic cardiomyopathy in perilipin-1 null mice with adipose tissue dysfunction. *Cardiovascular Research*.

[10] Lopaschuk G. D., Ussher J. R., Folmes C. D. L., Jaswal J. S., Stanley W. C. (2010). Myocardial fatty acid metabolism in health and disease. *Physiological Reviews*.

[11] Toda C., Diano S. (2014). Mitochondrial UCP2 in the central regulation of metabolism. *Best Practice & Research: Clinical Endocrinology & Metabolism*.

[12] Victorino V. J., Mencalha A. L., Panis C. (2015). Post-translational modifications disclose a dual role for redox stress in cardiovascular pathophysiology. *Life Sciences*.

[13] Cardoso S., Correia S., Carvalho C. (2014). Perspectives on mitochondrial uncoupling proteins-mediated neuroprotection. *Journal of Bioenergetics and Biomembranes*.

[14] Mailloux R. J., Harper M.-E. (2012). Mitochondrial proticity and ROS signaling: lessons from the uncoupling proteins. *Trends in Endocrinology and Metabolism*.

[15] Collins S., Pi J., Yehuda-Shnaidman E. (2012). Uncoupling and reactive oxygen species (ROS)—a double-edged sword for *β*-cell function? ‘moderation in all things’. *Best Practice & Research: Clinical Endocrinology & Metabolism*.

[16] Lee K.-U., Lee I. K., Han J. (2005). Effects of recombinant adenovirus-mediated uncoupling protein 2 overexpression on endothelial function and apoptosis. *Circulation Research*.

[17] Jin X., Xiang Z., Chen Y.-P., Ma K.-F., Ye Y.-F., Li Y.-M. (2013). Uncoupling protein and nonalcoholic fatty liver disease. *Chinese Medical Journal*.

[18] Ozcan C., Palmeri M., Horvath T. L., Russell K. S., Russell R. R. (2013). Role of uncoupling protein 3 in ischemia-reperfusion injury, arrhythmias, and preconditioning. *American Journal of Physiology—Heart and Circulatory Physiology*.

[19] Perrino C., Schiattarella G. G., Sannino A. (2013). Genetic deletion of uncoupling protein 3 exaggerates apoptotic cell death in the ischemic heart leading to heart failure. *Journal of the American Heart Association*.

[20] Petrosillo G., Ruggiero F. M., Paradies G. (2003). Role of reactive oxygen species and cardiolipin in the release of cytochrome c from mitochondria. *The FASEB Journal*.

[21] Jiang J., Huang Z., Zhao Q., Feng W., Belikova N. A., Kagan V. E. (2008). Interplay between bax, reactive oxygen species production, and cardiolipin oxidation during apoptosis. *Biochemical and Biophysical Research Communications*.

[22] Wu C.-C., Bratton S. B. (2013). Regulation of the intrinsic apoptosis pathway by reactive oxygen species. *Antioxidants & Redox Signaling*.

[23] Ricquier D., Bouillaud F. (2000). The uncoupling protein homologues: UCP1, UCP2, UCP3, StUCP and AtUCP. *The Biochemical Journal*.

[24] Berardi M. J., Shih W. M., Harrison S. C., Chou J. J. (2011). Mitochondrial uncoupling protein 2 structure determined by NMR molecular fragment searching. *Nature*.

[25] Hoang T., Matovic T., Parker J., Smith M. D., Jelokhani-Niaraki M. (2015). Role of positively charged residues of the second transmembrane domain in the ion transport activity and conformation of human uncoupling protein-2. *Biochemistry*.

[26] Ledesma A., García de Lacoba M., Rial E. (2002). The mitochondrial uncoupling proteins. *Genome Biology*.

[27] Nicholls D. G. (2001). A history of UCPI. *Biochemical Society Transactions*.

[28] Andrews Z. B., Diano S., Horvath T. L. (2005). Mitochondrial uncoupling proteins in the CNS: in support of function and survival. *Nature Reviews Neuroscience*.

[29] Woyda-Ploszczyca A. M., Jarmuszkiewicz W. (2014). Different effects of guanine nucleotides (GDP and GTP) on protein-mediated mitochondrial proton leak. *PLoS ONE*.

[30] Jiménez-Jiménez J., Ledesma A., Zaragoza P., González-Barroso M. M., Rial E. (2006). Fatty acid activation of the uncoupling proteins requires the presence of the central matrix loop from UCP1. *Biochimica et Biophysica Acta—Bioenergetics*.

[31] Echtay K. S., Murphy M. P., Smith R. A. J., Talbot D. A., Brand M. D. (2002). Superoxide activates mitochondrial uncoupling protein 2 from the matrix side. Studies using targeted antioxidants. *The Journal of Biological Chemistry*.

[32] Echtay K. S., Roussel D., St-Plerre J. (2002). Superoxide activates mitochondrial uncoupling proteins. *Nature*.

[33] Cadenas S., Buckingham J. A., Samec S. (1999). UCP2 and UCP3 rise in starved rat skeletal muscle but mitochondrial proton conductance is unchanged. *The FEBS Letters*.

[34] Buroker N. E., Young M. E., Wei C. (2007). The dominant negative thyroid hormone receptor *β*-mutant Δ337T alters PPAR*α* signaling in heart. *American Journal of Physiology—Endocrinology and Metabolism*.

[35] Krauss S., Zhang C.-Y., Lowell B. B. (2005). The mitochondrial uncoupling-protein homologues. *Nature Reviews Molecular Cell Biology*.

[36] Zhang M., Wang B., Ni Y.-H. (2006). Overexpression of uncoupling protein 4 promotes proliferation and inhibits apoptosis and differentiation of preadipocytes. *Life Sciences*.

[37] Chan S. L., Liu D., Kyriazis G. A., Bagsiyao P., Ouyang X., Mattson M. P. (2006). Mitochondrial uncoupling protein-4 regulates calcium homeostasis and sensitivity to store depletion-induced apoptosis in neural cells. *Journal of Biological Chemistry*.

[38] Akhmedov A. T., Rybin V., Marín-García J. (2015). Mitochondrial oxidative metabolism and uncoupling proteins in the failing heart. *Heart Failure Reviews*.

[39] Chong S. J. F., Low I. C. C., Pervaiz S. (2014). Mitochondrial ROS and involvement of Bcl-2 as a mitochondrial ROS regulator. *Mitochondrion*.

[40] Vozza A., Parisi G., De Leonardis F. (2014). UCP2 transports C4 metabolites out of mitochondria, regulating glucose and glutamine oxidation. *Proceedings of the National Academy of Sciences of the United States of America*.

[41] Pecqueur C., Bui T., Gelly C. (2008). Uncoupling protein-2 controls proliferation by promoting fatty acid oxidation and limiting glycolysis-derived pyruvate utilization. *The FASEB Journal*.

[42] McLeod C. J., Aziz A., Hoyt R. F., McCoy J. P., Sack M. N. (2005). Uncoupling proteins 2 and 3 function in concert to augment tolerance to cardiac ischemia. *Journal of Biological Chemistry*.

[43] Teshima Y., Akao M., Jones S. P., Marbán E. (2003). Uncoupling protein-2 overexpression inhibits mitochondrial death pathway in cardiomyocytes. *Circulation Research*.

[44] Ji X.-B., Li X.-R., Hao D. (2015). Inhibition of uncoupling protein 2 attenuates cardiac hypertrophy induced by transverse aortic constriction in mice. *Cellular Physiology and Biochemistry*.

[45] Cao W., Medvedev A. V., Daniel K. W., Collins S. (2001). *β*-Adrenergic activation of p38 MAP kinase in adipocytes: cAMP induction of the uncoupling protein 1 (UCP1) gene requires p38 MAP kinase. *The Journal of Biological Chemistry*.

[46] Villarroya F., Iglesias R., Giralt M. (2007). PPARs in the control of uncoupling proteins gene expression. *PPAR Research*.

[47] Barberá M. J., Schlüter A., Pedraza N., Iglesias R., Villarroya F., Giralt M. (2001). Peroxisome proliferator-activated receptor *α* activates transcription of the brown fat uncoupling protein-1 gene. A link between regulation of the thermogenic and lipid oxidation pathways in the brown fat cell. *The Journal of Biological Chemistry*.

[48] Pedraza N., Rosell M., Villarroya J. (2006). Developmental and tissue-specific involvement of peroxisome proliferator-activated receptor-*α* in the control of mouse uncoupling protein-3 gene expression. *Endocrinology*.

[49] Bugge A., Siersbæk M., Madsen M. S., Göndör A., Rougier C., Mandrup S. (2010). A novel intronic peroxisome proliferator-activated receptor *γ* enhancer in the Uncoupling Protein (UCP) 3 gene as a regulator of both UCP2 and -3 expression in adipocytes. *Journal of Biological Chemistry*.

[50] Baraldi F., Dalalio F., Teodoro B., Prado I., Curti C., Alberici L. (2014). Body energy metabolism and oxidative stress in mice supplemented with conjugated linoleic acid (CLA) associated to oleic acid. *Free Radical Biology and Medicine*.

[51] Solanes G., Pedraza N., Iglesias R., Giralt M., Villarroya F. (2003). Functional relationship between myod and peroxisome proliferator-activated receptor-dependent regulatory pathways in the control of the human uncoupling protein-3 gene transcription. *Molecular Endocrinology*.

[52] Van Der Lee K. A. J. M., Willemsen P. H. M., Van Der Vusse G. J., Van Bilsen M. (2000). Effects of fatty acids on uncoupling protein-2 expression in the rat heart. *The FASEB Journal*.

[53] Planavila A., Redondo-Angulo I., Ribas F. (2015). Fibroblast growth factor 21 protects the heart from oxidative stress. *Cardiovascular Research*.

[54] Perrino C., Schiattarella G. G., Sannino A. (2013). Genetic deletion of uncoupling protein 3 exaggerates apoptotic cell death in the ischemic heart leading to heart failure. *Journal of the American Heart Association*.

[55] Koziel A., Sobieraj I., Jarmuszkiewicz W. (2015). Increased activity of mitochondrial uncoupling protein 2 improves stress resistance in cultured endothelial cells exposed in vitro to high glucose levels. *American Journal of Physiology—Heart and Circulatory Physiology*.

[56] Chen Y., Liu J., Zheng Y. (2015). Uncoupling protein 3 mediates H_2_O_2_ preconditioning-afforded cardioprotection through the inhibition of MPTP opening. *Cardiovascular Research*.

[57] Nègre-Salvayre A., Hirtz C., Carrera G. (1997). A role for uncoupling protein-2 as a regulator of mitochondrial hydrogen peroxide generation. *The FASEB Journal*.

[58] Ruiz-Ramírez A., Chávez-Salgado M., Peñeda-Flores J. A., Zapata E., Masso F., El-Hafidi M. (2011). High-sucrose diet increases ROS generation, FFA accumulation, UCP2 level, and proton leak in liver mitochondria. *American Journal of Physiology—Endocrinology and Metabolism*.

[59] MacLellan J. D., Gerrits M. F., Gowing A., Smith P. J. S., Wheeler M. B., Harper M.-E. (2005). Physiological increases in uncoupling protein 3 augment fatty acid oxidation and decrease reactive oxygen species production without uncoupling respiration in muscle cells. *Diabetes*.

[60] Boudina S., Sena S., Theobald H. (2007). Mitochondrial energetics in the heart in obesity-related diabetes: direct evidence for increased uncoupled respiration and activation of uncoupling proteins. *Diabetes*.

[61] Brand M. D. (2010). The sites and topology of mitochondrial superoxide production. *Experimental Gerontology*.

[62] Koopman W. J. H., Distelmaier F., Smeitink J. A. M., Willems P. H. G. M. (2013). OXPHOS mutations and neurodegeneration. *The EMBO Journal*.

[63] Robbins D., Zhao Y. (2011). New aspects of mitochondrial uncoupling proteins (UCPs) and their roles in tumorigenesis. *International Journal of Molecular Sciences*.

[64] Lagouge M., Larsson N.-G. (2013). The role of mitochondrial DNA mutations and free radicals in disease and ageing. *Journal of Internal Medicine*.

[65] Antico Arciuch V. G., Elguero M. E., Poderoso J. J., Carreras M. C. (2012). Mitochondrial regulation of cell cycle and proliferation. *Antioxidants & Redox Signaling*.

[66] Tait S. W. G., Green D. R. (2012). Mitochondria and cell signalling. *Journal of Cell Science*.

[67] Orrenius S., Gogvadze V., Zhivotovsky B. (2007). Mitochondrial oxidative stress: implications for cell death. *Annual Review of Pharmacology and Toxicology*.

[68] Sverdlov A. L., Elezaby A., Qin F. (2016). Mitochondrial reactive oxygen species mediate cardiac structural, functional, and mitochondrial consequences of diet-induced metabolic heart disease. *Journal of the American Heart Association*.

[69] Chen Y.-R., Zweier J. L. (2014). Cardiac mitochondria and reactive oxygen species generation. *Circulation Research*.

[70] Ruiz-Ramírez A., Barrios-Maya M.-A., López-Acosta O., Molina-Ortiz D., El-Hafidi M. (2015). Cytochrome *c* release from rat liver mitochondria is compromised by increased saturated cardiolipin species induced by sucrose feeding. *American Journal of Physiology—Endocrinology and Metabolism*.

[71] Schwarzer M., Osterholt M., Lunkenbein A., Schrepper A., Amorim P., Doenst T. (2014). Mitochondrial reactive oxygen species production and respiratory complex activity in rats with pressure overload-induced heart failure. *Journal of Physiology*.

[72] Hang T., Huang Z., Jiang S. (2006). Apoptosis in pressure overload-induced cardiac hypertrophy is mediated, in part, by adenine nucleotide translocator-1. *Annals of Clinical and Laboratory Science*.

[73] Condorelli G., Morisco C., Stassi G. (1999). Increased cardiomyocyte apoptosis and changes in proapoptotic and antiapoptotic genes bax and bcl-2 during left ventricular adaptations to chronic pressure overload in the rat. *Circulation*.

[74] Shabalina I. G., Nedergaard J. (2011). Mitochondrial (‘mild’) uncoupling and ROS production: physiologically relevant or not?. *Biochemical Society Transactions*.

[75] Perry R. J., Zhang D., Zhang X.-M., Boyer J. L., Shulman G. I. (2015). Controlled-release mitochondrial protonophore reverses diabetes and protonophore reverses diabetes and steatohepatitis in rats. *Science*.

[76] Sluse F. E. (2012). Uncoupling proteins: molecular, functional, regulatory, physiological and pathological aspects. *Advances in Experimental Medicine and Biology*.

[77] Chen Y., Liu J., Zheng Y. (2015). Uncoupling protein 3 mediates H_2_O_2_ preconditioning-afforded cardioprotection through the inhibition of MPTP opening. *Cardiovascular Research*.

[78] Brennan J. P., Berry R. G., Baghai M., Duchen M. R., Shattock M. J. (2006). FCCP is cardioprotective at concentrations that cause mitochondrial oxidation without detectable depolarisation. *Cardiovascular Research*.

[79] Pucci G., Battista F., de Vuono S. (2014). Pericardial fat, insulin resistance, and left ventricular structure and function in morbid obesity. *Nutrition, Metabolism and Cardiovascular Diseases*.

[80] Sun X., Pan H., Tan H., Yu Y. (2012). High free fatty acids level related with cardiac dysfunction in obese rats. *Diabetes Research and Clinical Practice*.

[81] Neitzel A. S., Carley A. N., Severson D. L. (2003). Chylomicron and palmitate metabolism by perfused hearts from diabetic mice. *American Journal of Physiology—Endocrinology and Metabolism*.

[82] Zhou Y.-T., Grayburn P., Karim A. (2000). Lipotoxic heart disease in obese rats: implications for human obesity. *Proceedings of the National Academy of Sciences of the United States of America*.

[83] Granér M., Siren R., Nyman K. (2013). Cardiac steatosis associates with visceral obesity in nondiabetic obese men. *Journal of Clinical Endocrinology and Metabolism*.

[84] Tian Y.-Q., Li S.-S., Su X.-D. (2012). Effects of pioglitazone on high-fat-diet-induced ventricular remodeling and dysfunction in rats. *Journal of Cardiovascular Pharmacology and Therapeutics*.

[85] Schrammel A., Mussbacher M., Winkler S. (2013). Cardiac oxidative stress in a mouse model of neutral lipid storage disease. *Biochimica et Biophysica Acta—Molecular and Cell Biology of Lipids*.

[86] Carley A. N., Severson D. L. (2005). Fatty acid metabolism is enhanced in type 2 diabetic hearts. *Biochimica et Biophysica Acta (BBA)—Molecular and Cell Biology of Lipids*.

[87] Young M. E., Guthrie P. H., Razeghi P. (2002). Impaired long-chain fatty acid oxidation and contractile dysfunction in the obese Zucker rat heart. *Diabetes*.

[88] Martínez L., Torres S., Baulies A. (2015). Myristic acid potentiates palmitic acid-induced lipotoxicity and steatohepatitis associated with lipodystrophy by sustaning de novo ceramide synthesis. *Oncotarget*.

[89] Gao H., Feng X.-J., Li Z.-M. (2015). Downregulation of adipose triglyceride lipase promotes cardiomyocyte hypertrophy by triggering the accumulation of ceramides. *Archives of Biochemistry and Biophysics*.

[90] Wang S., Subramaniam A., Cawthorne M. A., Clapham J. C. (2003). Increased fatty acid oxidation in transgenic mice overexpressing UCP3 in skeletal muscle. *Diabetes, Obesity and Metabolism*.

[91] Stavinoha M. A., RaySpellicy J. W., Essop M. F. (2004). Evidence for mitochondrial thioesterase 1 as a peroxisome proliferator-activated receptor-*α*-regulated gene in cardiac and skeletal muscle. *American Journal of Physiology—Endocrinology and Metabolism*.

[92] Harmancey R., Vasquez H. G., Guthrie P. H., Taegtmeyer H. (2013). Decreased long-chain fatty acid oxidation impairs postischemic recovery of the insulin-resistant rat heart. *The FASEB Journal*.

[93] Boardman N. T., Larsen T. S., Severson D. L., Essop M. F., Aasum E. (2011). Chronic and acute exposure of mouse hearts to fatty acids increases oxygen cost of excitation-contraction coupling. *American Journal of Physiology—Heart and Circulatory Physiology*.

[94] Costford S. R., Chaudhry S. N., Crawford S. A., Salkhordeh M., Harper M.-E. (2008). Long-term high-fat feeding induces greater fat storage in mice lacking UCP3. *American Journal of Physiology—Endocrinology and Metabolism*.

[95] Mazumder P. K., O'Neill B. T., Roberts M. W. (2004). Impaired cardiac efficiency and increased fatty acid oxidation in insulin-resistant ob/ob mouse hearts. *Diabetes*.

[96] Kong J. Y., Rabkin S. W. (2002). Palmitate-induced cardiac apoptosis is mediated through CPT-1 but not influenced by glucose and insulin. *American Journal of Physiology—Heart and Circulatory Physiology*.

[97] Murray M., Dyari H. R. E., Allison S. E., Rawling T. (2014). Lipid analogues as potential drugs for the regulation of mitochondrial cell death. *British Journal of Pharmacology*.

[98] Di Paola M., Cocco T., Lorusso M. (2000). Arachidonic acid causes cytochrome c release from heart mitochondria. *Biochemical and Biophysical Research Communications*.

[99] Schönfeld P., Wojtczak L. (2007). Fatty acids decrease mitochondrial generation of reactive oxygen species at the reverse electron transport but increase it at the forward transport. *Biochimica et Biophysica Acta (BBA)—Bioenergetics*.

[100] Seifert E. L., Bézaire V., Estey C., Harper M.-E. (2008). Essential role for uncoupling protein-3 in mitochondrial adaptation to fasting but not in fatty acid oxidation or fatty acid anion export. *The Journal of Biological Chemistry*.

[101] Schrauwen P., Hoeks J., Schaart G. (2003). Uncoupling protein 3 as a mitochondrial fatty acid anion exporter. *The FASEB Journal*.

[102] Echtay K. S., Esteves T. C., Pakay J. L. (2003). A signalling role for 4-hydroxy-2-nonenal in regulation of mitochondrial uncoupling. *The EMBO Journal*.

[103] Brand M. D., Pamplona R., Portero-Otín M. (2002). Oxidative damage and phospholipid fatty acyl composition in skeletal muscle mitochondria from mice underexpressing or overexpressing uncoupling protein 3. *Biochemical Journal*.

[104] Dyntar D., Eppenberger-Eberhardt M., Maedler K. (2001). Glucose and palmitic acid induce degeneration of myofibrils and modulate apoptosis in rat adult cardiomyocytes. *Diabetes*.

[105] Ostrander D. B., Sparagna G. C., Amoscato A. A., McMillin J. B., Dowhan W. (2001). Decreased cardiolipin synthesis corresponds with cytochrome c release in palmitate-induced cardiomyocyte apoptosis. *Journal of Biological Chemistry*.

[106] Teshima Y., Akao M., Jones S. P., Marbán E. (2003). Uncoupling protein-2 overexpression inhibits mitochondrial death pathway in cardiomyocytes. *Circulation Research*.

[107] Bosma M., Dapito D. H., Drosatos-Tampakaki Z. (2014). Sequestration of fatty acids in triglycerides prevents endoplasmic reticulum stress in an in vitro model of cardiomyocyte lipotoxicity. *Biochimica et Biophysica Acta (BBA)—Molecular and Cell Biology of Lipids*.

[108] Kelly D. P., Hale D. E., Rutledge S. L. (1992). Molecular basis of inherited medium-chain acyl-CoA dehydrogenase deficiency causing sudden child death. *Journal of Inherited Metabolic Disease*.

[109] Kurtz D. M., Rinaldo P., Rhead W. J. (1998). Targeted disruption of mouse long-chain acyl-CoA dehydrogenase gene reveals crucial roles for fatty acid oxidation. *Proceedings of the National Academy of Sciences of the United States of America*.

[110] Pagano C., Calcagno A., Granzotto M. (2008). Heart lipid accumulation in obese non-diabetic rats: effect of weight loss. *Nutrition, Metabolism and Cardiovascular Diseases*.

[111] Roussel J., Thireau J., Brenner C. (2015). Palmitoyl-carnitine increases RyR2 oxidation and sarcoplasmic reticulum Ca^2+^ leak in cardiomyocytes: role of adenine nucleotide translocase. *Biochimica et Biophysica Acta—Molecular Basis of Disease*.

[112] Fauconnier J., Andersson D. C., Zhang S.-J. (2007). Effects of palmitate on Ca^2+^ handling in adult control and ob/ob cardiomyocytes: impact of mitochondrial reactive oxygen species. *Diabetes*.

[113] Alrob O. A., Lopaschuk G. D. (2014). Role of CoA and acetyl-CoA in regulating cardiac fatty acid and glucose oxidation. *Biochemical Society Transactions*.

[114] Hirschey M. D., Shimazu T., Jing E. (2011). SIRT3 deficiency and mitochondrial protein hyperacetylation accelerate the development of the metabolic syndrome. *Molecular Cell*.

[115] Bharathi S. S., Zhang Y., Mohsen A.-W. (2013). Sirtuin 3 (SIRT3) protein regulates long-chain acyl-CoA dehydrogenase by deacetylating conserved lysines near the active site. *The Journal of Biological Chemistry*.

[116] Hirschey M. D., Shimazu T., Goetzman E. (2010). SIRT3 regulates mitochondrial fatty-acid oxidation by reversible enzyme deacetylation. *Nature*.

